# Pathogenesis and virulence of flavivirus infections

**DOI:** 10.1080/21505594.2021.1996059

**Published:** 2021-11-22

**Authors:** Sophie Wilhelmina van Leur, Tiaan Heunis, Deeksha Munnur, Sumana Sanyal

**Affiliations:** Sir William Dunn School of Pathology, University of Oxford, South Parks Road, Oxford OX1 3RE, UK

**Keywords:** Flavivirus, pathogenesis, life-cycle, immunity, Dengue, Zika, host-pathogen interactions

## Abstract

The Flavivirus genus consists of >70 members including several that are considered significant human pathogens. Flaviviruses display a broad spectrum of diseases that can be roughly categorised into two phenotypes – systemic disease involving haemorrhage exemplified by dengue and yellow Fever virus, and neurological complications associated with the likes of West Nile and Zika viruses. Attempts to develop vaccines have been variably successful against some. Besides, mosquito-borne flaviviruses can be vertically transmitted in the arthropods, enabling long term persistence and the possibility of re-emergence. Therefore, developing strategies to combat disease is imperative even if vaccines become available. The cellular interactions of flaviviruses with their human hosts are key to establishing the viral lifecycle on the one hand, and activation of host immunity on the other. The latter should ideally eradicate infection, but often leads to immunopathological and neurological consequences. In this review, we use Dengue and Zika viruses to discuss what we have learned about the cellular and molecular determinants of the viral lifecycle and the accompanying immunopathology, while highlighting current knowledge gaps which need to be addressed in future studies.

## Graphical Abstract

**Figure:**
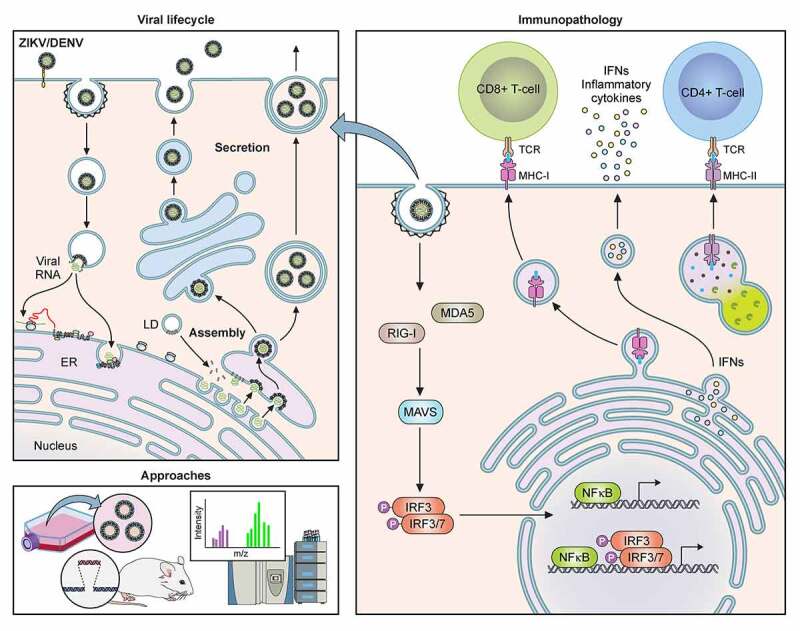

## Introduction

Flaviviruses are positive sense single-stranded RNA viruses transmitted predominantly by arthropods and includes some of the most important human pathogens such as dengue (DENV1-4), yellow fever (YFV), West Nile (WNV) and Zika (ZIKV) viruses. Flaviviruses display a wide spectrum of clinical manifestations ranging from asymptomatic or mild fever to haemorrhagic disease as observed in dengue and yellow fever or encephalitic disease in ZIKV and Japanese Encephalitis (JEV) infections[1]. These pathogens pose a significant burden of disease with dengue alone being responsible for ~400 million infections and 100 million symptomatic cases every year[2]. Prophylactic vaccinations have been successful for some flaviviruses such as Japanese encephalitis virus (JEV) and YFV. In case of YFV, however, epidemics continue to occur by re-establishment of the vector in human populations due to globalization and urbanization [3,4]. Apart from the extensive geographical spread of the vectors (predominantly the *aedes* species of mosquitos) and increased vector contact with human populations, a major factor contributing to the global threat of flavivirus infections, is the current lack of effective, FDA-approved antivirals[5]. It is therefore imperative to comprehensively understand the biology of flaviviruses.

Flaviviruses are enveloped RNA viruses (11kb size, ~50 nm in diameter) with an electron-dense spherical nucleocapsid core (~30 nm), comprising multiple capsid (C) copies in complex with the single stranded RNA molecule[6]. It encodes for 3 structural proteins (capsid; C, pre-membrane; prM and Envelope; E) and seven non-structural proteins (NS1, NS2A, NS2B, NS3, NS4A, NS4B, NS5). The nucleocapsid is enclosed within a host-cell derived lipid bilayer, which anchors 180 copies of the other two structural proteins, M and E in a mature virion. The bilayer has an icosahedral envelope organization, with the 90 E dimers organized into a metastable “herringbone” configuration [7–9]. The structural heterogeneity allows for “breathing” and has been suggested to allow for broader flaviviral host-range and tissue tropism by alternative receptor interactions but most likely plays distinct roles in different stages of the viral life cycle as described below [9,10].

## Viral life cycle

### Viral binding and entry

**Figure 1.**
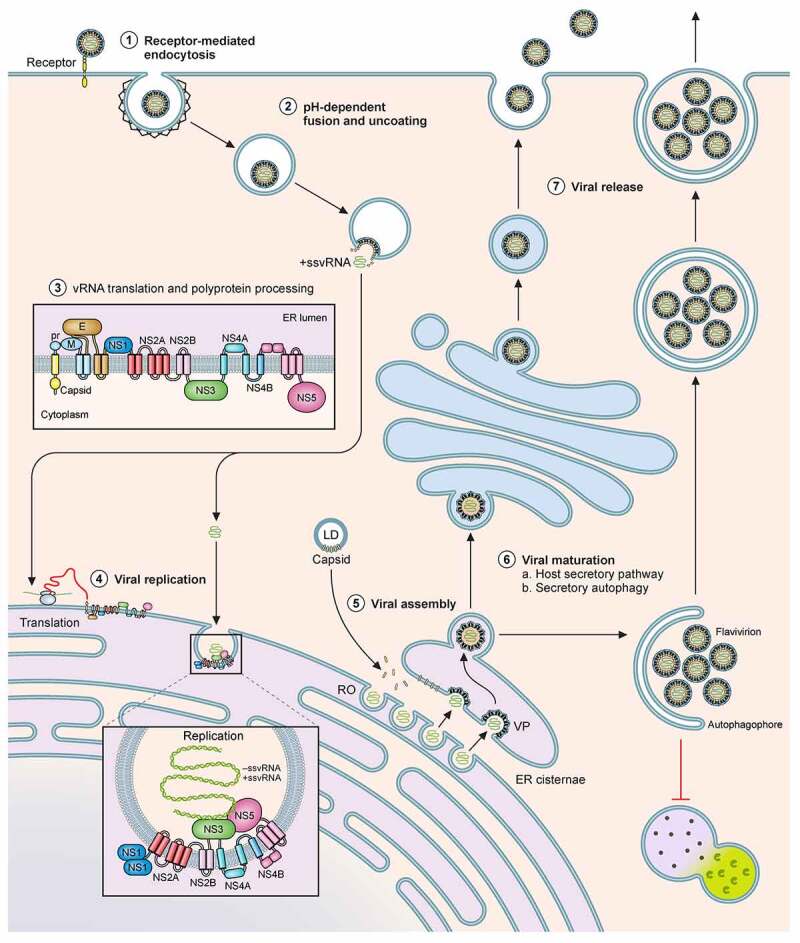
**Schematic of the flavivral lifecycle**. The Flaviviral lifecycle starts with the (1) internalization of the virion by attachment and receptor-mediated endocytosis. (2) Under the mildly acidic environment of the endosome, the viral membrane fuses with that of the endosomal membrane, uncoats and the nucleocapsid is released into the cytoplasm. (3) In the cytoplasm, vRNA is translated into a polyprotein by the host translational machinery and inserted into the ER membrane. The polyprotein is subsequently cleaved by host and viral proteins to generate 3 structural proteins and at least 7 non-structural proteins. (4) These non-structural proteins form the viral replication complex. After initial viral replication, the viral proteins assist in generating Replication Organelles (RO) and vesicle packets (VP) in the ER shielding the virus from the cytoplasm. In the RO, +ssRNA is generated and transported to another invagination in the ER, the assembly site, the VP. (5) For encapsidation of the +ssRNA, capsid protein accumulates and complexes on lipid droplets (LD) and is recruited to the VP alongside the +ssRNA. Heterodimers of prM and E are recruited to the VP and trimerize on the membrane. Subsequently the VP can bud into the ER forming an immature virion. (6) Virions accumulate in ER cisternae before transport for maturation through the GBF1-dependent host secretory pathway or through secretory autophagy. (7) The virus can be released as free virions or additionally, in membrane enclosed forms derived from autophagosomes, in a host-derived lipid bilayer as quasispecies

The infectious life cycle of flaviviruses can be roughly divided into stages of binding and entry, translation and replication, assembly and release (Figure 1). For binding and entry, the virus particles first attach to the cell surface; subsequently, the viral glycoprotein E binds to a cellular receptor to initiate internalization via endocytosis. The specific entry receptor required for internalization of individual members of flaviviruses are yet to be identified, but these putative receptors are expected to be a widely expressed, given the wide range of cell types of human, hamster, murine and monkey origin that are permissive to infection [11]. Recent advances in mass spectrometry and proteomics strategies are anticipated to aid in the discovery of such receptors (see section 5.1.1 (Mass spectrometry based approaches)). Several ubiquitously expressed cell surface markers have been identified as attachment factors [12]. Low-affinity attachment factors such as negatively charged glycosaminoglycans, e.g., heparin sulfate and C-type lectins DC-SIGN (dendritic cell-specific intracellular adhesion molecule-3-grabbing non-integrin), have been proposed to help concentrate virions through the DIII domain of glycoprotein E [13,14]. Stress-induced attachment factors such as heat-shock proteins 90 and 70 and the ER chaperone BiP/GRP78 have been shown to assist entry of DENV1, DENV2, and ZIKV [15,16]. In addition, phosphatidylserine receptors have also been reported to serve as entry factors for dengue virus [17]. After receptor or receptor-complex recognition, endocytosis is thought to occur primarily in clathrin-coated pits, although clathrin-independent calveolae/cholesterol-dependent entry mechanisms have also been proposed for JEV [18]. Endocytosis is completed upon dynamin-dependent scission [19]. These virions within endosomal compartments undergo viral uncoating triggered by the mildly acidic environment, thus enabling fusion of the viral envelope with the endosomal membrane [20,21]. Depending on the pH threshold of the flaviviral E glycoprotein and potentially the lipid composition of membranes, the virions can fuse with distinct endosomal compartments, either in early Rab5-positive, late Rab7-positive endosomes or in perinuclear endosomes, as seen for DENV serotypes [22].

### Translation, replication, and assembly

Upon fusion, the positive-strand RNA genome is released into the cytoplasm and translated for synthesis of the viral polyprotein, which is cleaved by host and viral proteases into the structural proteins C, prM and E and the non-structural proteins NS1, NS2A/B, NS3, NS4A/B, and NS5. Synthesis of the polyprotein occurs at the endoplasmic reticulum (ER), and apart from the co-translational translocation components, is also dependent on the ER membrane protein complex (EMC) for correct topology and stable expression [23,24].

The viral non-structural proteins in concert with host proteins orchestrate massive rearrangements to form convoluted membranes and invaginations in the ER referred to as replication organelles (RO) [25,26]. Here, the positive-sense RNA is used as a template for the viral polymerase NS5 to generate intermediate negative-sense RNA, which then serve to produce positive sense progeny RNAs for either incorporation into newly formed virus particles or for translation [26,27].

Progeny viral RNA is encapsulated into immature virions in vesicle packets (VPs) formed along the rough ER, in close proximity to the ROs. Recent reports indicate that atlastins, a subset of ER proteins, serve as central hubs to induce membrane remodelling and drive RO formation, virus replication and assembly [28]. The latter steps are tightly coordinated and VPs often appear juxtaposed to the ROs to enable direct encapsulation of the newly synthesized RNA [27,29,30]. Newly translated capsid protein is recruited to the VP sites on lipid droplets to encapsidate viral RNA into the nucleocapsid. Alongside, the structural prM and E proteins are recruited to the VPs as heterodimers facing the ER lumen. They subsequently form a trimer to assist budding of the nucleocapsid into the ER. Viral NS3 has been proposed to recruit the host endosomal sorting complexes for transport (ESCRT) machinery to drive fission of the membrane invaginations and release immature virions into the ER [31,32], detectable by transmission electron microscopy, prior to their transport and secretion into the extracellular space [25,26].

Biogenesis of the ROs and VPs in the ER is tightly regulated by the non-structural viral proteins, both to prevent innate immune activation and ER stress and to generate the requisite energy and membrane resources. For efficient reorganization cellular lipid metabolism is usurped by the viruses and cholesterol and fatty acid synthesis are locally established at the sites of replication and assembly [33,34]. Several viral proteins have been suggested to help in orchestrating this compartmentalization. First, the NS1 protein of DENV and ZIKV has been shown to orchestrate the invagination process of the RO and VPs [35,36]. In addition, local cholesterol synthesis and fatty acid synthesis are required and established by the non-structural flaviviral proteins. Replication is severely impaired upon inhibiting these pathways in primary human monocytes and other cell lines [34,37]. However, current understanding on how these metabolic pathways are exploited is still limited. For DENV, the non-structural protein NS3 was reported to modulate fatty acid metabolism by direct recruitment of the key enzyme, fatty acid synthase (FASN) to the replication sites via Rab18 to increase fatty acid synthesis in proximity to viral RNA [38,39]. Similarly, increased fatty acid synthase activity was also observed in ZIKV placental infection[40].

For local cholesterol biosynthesis, specific recruitment of 3-hydroxy-3-methylglutaryl-CoA reductase (HMGCR), the rate-limiting enzyme in this pathway has been proposed for several flaviviruses. For WNV, HMGCR was clearly shown to be recruited to virus-induced VPs and ROs along with general redistribution of cholesterol to these sites[37]. Moreover, DENV NS4A was reported to specifically co-localize with HMGCR at these sites[41].

Besides lipogenesis, the non-structural transmembrane protein complex of NS4A and NS4B was reported to assist in ER remodelling by induction of lipophagy (selective degradation of lipid droplets), as seen for DENV [42]. The complex was shown to recruit host protein Aup1 and hijack its acyltransferase activity to induce lipophagy [43]. The energy and free fatty acids generated in lipophagy have previously been suggested as essential for flaviviral replication [44]. More recent work has led to the development in experimental tools such as a plasmid-based replication-independent system of generating replication organelles [45]. Using this system, the 3ʹ terminal RNA region of DENV, but not ZIKV was reported to contribute to the biogenesis of ROs [46].

### Maturation and secretion

Our current understanding of the final steps in flaviviral assembly and secretion is limited. Multiple exit strategies have been proposed. The immature virus progenies accumulate in the ER cisternae as large cargo, which are believed to be transported along the host secretory pathway for virion maturation. Immature virions undergo furin-mediated prM cleavage, N-linked glycosylation and ubiquitylation of the E-protein, which are acquired *en route* to the trans-Golgi [47–49]. DENV1-3 particles have been shown to recruit KDEL receptors to traffic from the ER and arrive at the Golgi in a Golgi-specific Brefeldin A-resistant guanine nucleotide exchange factor 1 (GBF1)-dependent manner within large cargo-specific vesicles [50]. After undergoing maturation, viral progenies exit the cell via currently undefined strategies. EM studies show that virus particles can be detected close to or in the process of fusing with the plasma membrane in a multitude of vesicles including those containing a single virion as well as those carrying vesicles packaged within membrane-enclosed structures [51]. The latter can be linked to the secretory arm of autophagy [52] – an alternative exit route suggested for flaviviruses [53]. Inhibition of autophagy was reported to abolish prM cleavage of DENV virions [54]. This pathway might thus provide an alternative route for maturation by its mildly acidic environment. Specifically the noncanonical secretory arm of this pathway was linked to DENV maturation as inhibiting lysosomal fusion increased virus titres [54]. Secretory amphisomes generated in DENV-infected cells were proposed to contain virions, since extracellular vesicles were detected in DENV-infected human serum and in Huh7 cells, containing lipid droplet and autophagic markers alongside viral RNA, prM/M and E [55]. Furthermore, dsRNA and NS1 was found to co-localize with LC3, initially leading to the hypothesis that alternative DENV2 replication could occur on or within amphisomes derived from the autophagy pathway [56]. Later, the amphisomal compartments were linked to assembly and secretion rather than replication for DENV and ZIKV, as impairment of autophagosome formation had minimal influence on viral replication while attenuating viral titres [54,57]. Interestingly, both DENV and ZIKV are able to utilize ATG5-independent LC3 lipidation, but ZIKV alone is dependent on both autophagosome initiation complexes ULK1/FIP200 and VPS34/BECN1, whereas DENV is only dependent on VPS34. How these virus-containing amphisomes are targeted for release rather than degradation remains unknown, but recent studies have provided additional insights into how these structures can be trafficked for release [53]. The amphisome-dependent exit strategy of DENV was linked to manipulation of the Src family kinases (SFKs). In this study, Lyn-dependent egress of virus particles was observed where LC3+ secretory organelles enabled bulk viral release in a Rab11-dependent manner [58]. Such alternative routes can affect tissue tropism and possibly function as immune evasion strategy for viruses, to escape detection by circulating antibodies and manipulate cell-to-cell communication [55,58,59]. This non canonical secretory route has also been suggested to contribute to transplacental spread of ZIKV[60]. Recent studies have also indicated that the released host phosphatidylethanolamine lipids (PE) might play a role in stabilizing ZIKV and DENV2 virions for release by filling a lipid-binding pocket generated during maturation [61,62]. Considering infectious virus can be detected for ZIKV in urine, semen and vaginal fluids, virion stability is a necessity for flaviviral transmission and host lipids could thus play a key role. Several outstanding questions remain to be addressed in the viral lifecycle, not the least of which is the mechanism of biogenesis of ROs. In particular in vitro reconstitutions using liposomes supplemented with host and viral factors to generate morphologically similar ROs as observed in infected samples are necessary to provide key insights that may well be relevant for other +RNA viruses.

## Immune responses to flavivirus infection

### Innate immune activation

**Figure 2.**
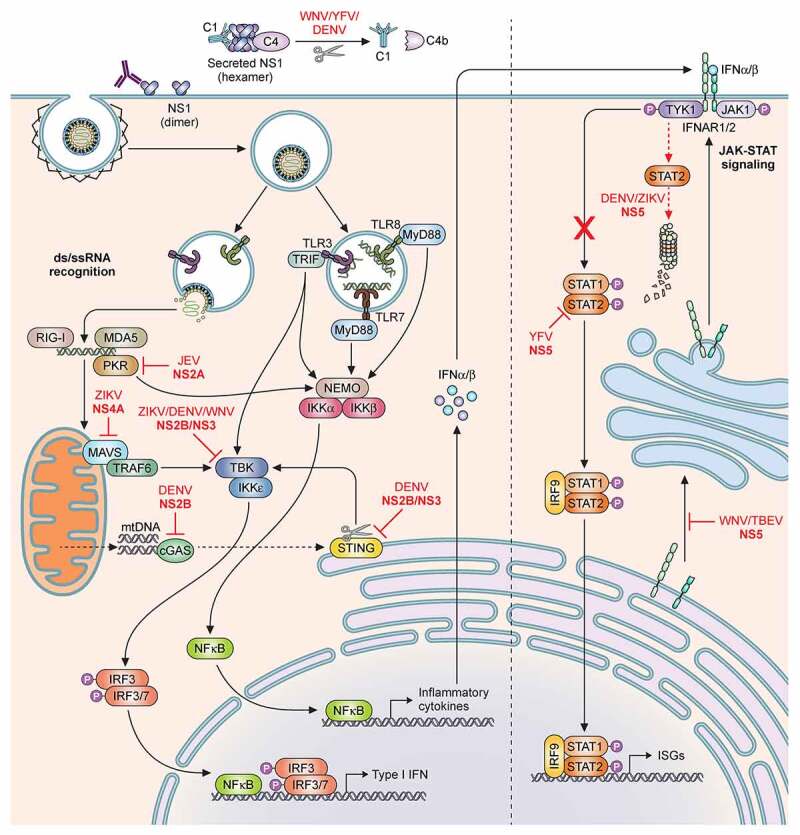
**Schematic of the host innate immune response and flaviviral antagonism**. Innate immune activation starts by recognition of flaviviral ds- or ssRNA either in the cytoplasm by RLRs; MDA5, RIG-I, PKR; or in the endosomes by TLRs; TLR3, TLR7 and TLR8. Subsequently the NFκB pathway and IRF pathways can be activated to initiate the production of Type I IFN and a range of inflammatory cytokines. The secreted IFNα and IFNβ can subsequently initiate the JAK-STAT signalling cascade to induce ISGs. Flaviviruses have developed multiple routes of intervention in both pathways. The viral non-structural protein NS2A can interact with PKR (YFV) and TBK (ZIKV/DENV) and block signalling. Similarly, NS4B of ZIKV, WNV and DENV serotypes 1,2,4 can prevent phosphorylation of TBK. NS4A of ZIKV is able to antagonize the MAVS signalling pathway by binding MAVS. NS2B/NS3 cleaves STING to prevent mitochondrial DNA-induced Type I IFN production. In the same pathway NS2B of DENV can interact with cGAS and prevent mtDNA recognition. The protease NS5 intervenes in JAK-STAT signalling by either preventing ISGylation of STAT2 (YFV); by inducing degradation of STAT2 (DENV/ZIKV); or by suppressing its maturation (WNV/TBEV). Additionally, secreted hexameric NS1 of WNV, YFV, DENV, can induce Complement factor C4 degradation and thus complement activation

As described in section 1 (viral life-cycle), the flavivirus life-cycle is confined within the host cell cytoplasm. The host cell is equipped with pattern recognition receptors (PRRs) distributed within endosomal compartments and the cytoplasm, which are able to detect and respond to viral RNA and virus-infected cells. The innate immune response is the first line of defence against invading pathogens[63]; it is rapid and non-specific but plays a key role in establishing adaptive immunity, which is pathogen-specific and provides long-lasting immunological memory [64,65]. There are two events that are required to trigger effective immune response – a) detection of invading virus molecules by specialised sensors, the PRRs, and b) PRRs further activate protein signalling cascades that regulate synthesis and secretion of type I interferons (IFNs). PRRs can recognise pathogen-associated molecular patterns (PAMPs) such as double-stranded RNA (dsRNA), single-stranded RNA (ssRNA) and demethylated CpG rich motifs (CpGDNA). There are 3 major classes of PRRs that partake in efficient detection of flaviviruses – a) toll-like receptor (TLR), b) retinoic acid-inducible gene I (RIG-I) – like receptors and c) cyclic GMP-AMP synthase (cGAS) (Figure 2).

#### Toll-like receptors (TLR)

TLRs were one of the earliest groups of PRRs recognised. The TLR family comprises 10 members in humans, TLR1-TLR10. TLRs localise to the cell surface or intracellular compartments such as ER, endosomes, or lysosomes and sense viral RNA. Flavivirus RNA present in endosomes are recognised mainly by TLR3 and TLR7 [66,67]. Most of the available information on the role of TLRs in flavivirus infection emerged from studies with WNV where TLR3 and TLR7 were reported to promote CNS immunity and restrict virus replication. Secreted WNV NS1 was able to antagonise the TLR3-dependent antiviral response [68]. Further studies have shown TLR3 to be critical for detection and control of both DENV and ZIKV. Knockout of TLR3 in macrophages resulted in increased susceptibility to DENV infection [69] while knockdown of TLR3 in skin fibroblasts increased ZIKV viral RNA, although overall IFN-I mRNA levels were not affected. Interestingly, in cells of the nervous system, which are often targets of ZIKV infection, activation of TLR3 increased the inflammatory response while dampening the antiviral interferon effect mounted via the RIG-I signalling cascade, thereby complicating the immunopathology of infection [70]. A screen to identify therapeutic molecules against ZIKV revealed R848 (resiquimod) as a potent inhibitor of ZIKV replication in monocytes and macrophages. Given that R848 functions as a TLR7/8 agonist suggested that these TLRs might also contribute to restricting infection. R848 treatment induced expression of several antiviral genes, most prominently Viperin, that was proposed to restrict ZIKV in microglia and macrophages [71].

#### Retinoic acid inducible gene-I like receptors (RLRs)

The RLRs are cytosolic PRRs that recognise non-self RNA (typically dsRNA) and upon interaction with the mitochondrial antiviral signalling protein (MAVS) trigger activation of interferons, inflammatory cytokines and further downstream expression of interferon stimulated genes [72]. This family of PRRs consists of RIG-I, melanoma differentiation-associated protein 5 (MDA5) and LGP2. Both RIG-I and MDA5 are critical for immune response against flaviviruses. Most of the established data emerged from studies with WNV [73,74]; however, recent studies have also indicated their involvement in mounting an immune response to DENV and ZIKV upon recognition of the 5ʹ end of the viral RNA [75]. MAVS knockout mice also display increased levels of DENV RNA and delayed production of IFNs [76].

#### cGAS/STING

In addition to producing dsRNA, flaviviruses are known to release mitochondrial DNA (mtDNA) into host cells during infection, most likely mediated by IL-1β[77]. The host immune system has developed strategies to detect viral DNA particles via the cytosolic DNA sensing pathway. Some of the well-characterised DNA sensors that play an important role in innate immune response are TLR9, IFN-γ-inducible protein 16 (IFI16), absent in melanoma 2 (AIM2), DEAD box polypeptide 41 (DDX41), and cGAS [78]. The cGAS/stimulator of interferon genes (STING) pathway is one of the principal cytosolic DNA sensing system that has been implicated in flavivirus restriction [77]. Knockout of cGAS in THP-1 cells display increased ZIKV replication and decreased host resistance. Similarly, an IL-1β-cGas/STING-IRF3 innate immune program was found to be critical for dengue infection control [77].

#### Type-I IFN and IFN signalling in flavivirus infections

Each of the above mentioned viral nucleic acid sensors – TLR, RLR, cGAS eventually leads to transcriptional upregulation of hundreds of immunity factors such as IFNα, IFNβ and other IFN subtypes in an IRF (IRF3 & IRF7) and NF-κβ dependent manner. Secreted type I IFNs bind to IFNAR1/2 causing hetero-dimerization of receptor subunits and activating the janus kinase 1 (JAK1) STAT signalling cascade. Phosphorylation of cytosolic STAT1 and STAT2 causes hetero-dimerization and nuclear translocation followed by binding to IRF9 to form the heterotrimeric complex Interferon stimulated gene factor 3 (ISGF3), which in turn binds the IFN-stimulated response element to trigger expression of >100 IFN-stimulated genes (ISGs) creating an antiviral state. ISGs play an important role in influencing cellular antiviral mechanisms including RNA processing, protein turnover and apoptosis thus directly affecting specific stages of virus life cycle and replication. Some ISGs implicated in inhibiting DENV and/or ZIKV infection include – ISG15, interferon-inducible transmembrane family (IFITM2 and IFITM3), interferon alpha-inducible protein family (IFI6 and IFI16), virus inhibiting protein endoplasmic reticulum associated interferon induced (Viperin), ArfGAP with dual pleckstrin homology (PH) domain2 (ADAP2), tripartite motif protein family (TRIM19, TRIM56 and TRIM69), OAS1 (2ʹ, 5ʹ-oligoadenylate synthetase), RyDEN (repressor of yield of DENV), FMRP (Fragile X mental retardation protein), IFIT1-3 (IFB-induced protein with tetratricopeptide repeats family), and MX1 (myxovirus resistance 1). These ISGs have been reviewed extensively elsewhere [79]. IFN-I treatment strongly inhibits DENV and ZIKV infection [65,80,81]. Mice lacking IFNAR1 were reported to be highly sensitive to DENV infection [82]. Control of DENV infection via IFN-mediated response involves both STAT1-dependent and the STAT1-independent pathways, where the former controls the initial viral burden and latter resolves DENV infection in mice [83]. Mice deficient in IFNAR1, IFNAR2 and STAT2 are also more susceptible to ZIKV infection compared to WT mice [84,85]. Mice lacking INFAR1 or IRF3/5/7 triple knockout mice were reported to develop neurological defects and succumb to ZIKV infection [86].

#### Viral antagonism of innate immune responses

The initial immune barriers that viruses have to overcome to establish a successful infection includes type-I interferons, inflammatory cytokines, complement response, NK cell immunity, apoptosis and autophagy. To subvert these pathways, flaviviruses have evolved several strategies. Viral NS1 of WNV, YFV and DENV were shown to antagonize complement activation [87]. NS1 glycosylation appears to be a key determinant for its stability and secretion, in order to efficiently interact with and prevent complement activation [87]. Dampening of IFN-I was reported to occur via NS4B of flaviviruses [88]. NS2B was shown to target cGAS to prevent sensing of mtDNA, an indicator of cell damage [89]. Further studies have indicated that the host range of ZIKV is dependent on the viral NS2B3 proteolytic cleavage of STING [90,91]. CRISPR/Cas9 mediated knockout of STING in human and murine cell lines increased susceptibility of mouse but not human to ZIKV infection [75]. DENV replication is enhanced in both human and mouse primary cells lacking STING [89], where the NS2B/NS3 protease blocks type I IFN signalling by cleaving STING [91]. The best studied mechanism is NS5-dependent antagonism of JAK-STAT signalling. NS5 is able to induce STAT2 degradation, as seen for ZIKV and DENV [85], or prevent its ISGylation as observed in YFV [92]. WNV and Tick-borne encephalitis virus (TBEV) alternatively inhibit the cascade by suppressing IFNAR maturation and expression [93]. Another cellular pathway extensively manipulated by DENV and ZIKV is autophagy, which has been reviewed in detail elsewhere [94].

### Adaptive immune responses

Adaptive immunity to DENV and ZIKV is largely complicated by the heterogeneity of extracellular populations of viral particles, further complicated by the cross-reactivity of antibodies and T-cell responses to previous exposure of structurally similar virus strains. Apart from the different serotypes of dengue itself, the high degree of homology between DENV and ZIKV can also lead to severe disease outcomes. The characteristics of released particles can vary dramatically based on cell-type, insect versus mammalian host, temperature and the initial dose of the inoculum [95]. Proteolytic cleavage of prME is typically inefficient and often results in a mix of mature and immature particles with a wide spectrum of partially mature versions. Virions produced in insect cells have glycosylation patterns on their structural proteins, in particular, untrimmed terminal mannose residues are present in virions produced from insect cells whereas those produced from mammalian cells have additional sialic acids and other complex glycans on trimmed mannoses. Particles with a higher prM content is produced by insect cells versus mammalian dendritic cells. All these factors may contribute to some extent to the variability in immune responses [96,97].

#### T cell responses against flaviviruses

Individuals infected with DENV or immunized with the currently licensed, live attenuated tetravalent vaccine mount T cell responses to epitopes that covers majority of the DENV sequence. The CD8^+^ T cells preferentially target DENV NS3, NS5, and NS4B, while CD4^+^ T cells target structural proteins and NS1 [98,99]. Additional studies have indicated that robust DENV peptide-specific T cell responses, in particular to NS3 is correlated with significantly reduced viraemia [100]. The frequency of NS3, NS1 and NS5 peptide specific T cell responses determined in 74 patients with acute dengue infection revealed that both total and NS3-specific responses were found to be higher in patients with mild disease compared to those with severe pathology, indicative of the protective role of T cells [100]. Similarly, differences in CD8+ and CD4+ epitope targets have also been noted in JEV infection and for live YFV immunization [101,102]. Among JEV infected patients, CD4+ responses were associated with complete recovery. CD8+ responses primarily targeted non-structural proteins whereas CD4+ cells targeted the structural proteins and NS1 [96]. As with DENV, ZIKV-infected patients also display T cell responses to epitopes that cover the entire viral proteome. CD8^+^ T cells preferentially target structural proteins, whereas CD4^+^ T cells target both structural and non-structural proteins. However, in case of prior exposure to DENV, the distribution of CD8+ T cells was skewed towards the non-structural proteins of ZIKV [103]. When tested in a mouse model, ZIKV-infection elicited CD8+ T cell responses targeting prM, E and NS5, partially recapitulating the clinical features. In line with previous studies CD8+ T-cells were found to be protective – transfer of ZIKV-immune CD8+ T cell reduced, while depletion increased the viral load in mice [104].

Flaviviruses share significant sequence identities, which often leads to cross-reactive responses that can either boost protection or exacerbate the disease during a secondary infection with a related strain. In a process referred to as original antigenic sin, sequential infections with related viruses results in the subsequent response – be it antibodies or T cells, to be driven by less potent memory cells from the primary exposure. As a result, low-avidity clones are generated, which in turn mount an ineffective immune response often leading to immunopathology. This is often the case with DENV infections – the onset of severe dengue symptoms with aberrant T cell activation and a “cytokine storm” suggests that T cells may induce immunopathology in severe disease [98,105,106]. In a study performed with a cohort of Thai children, very few dengue responsive CD8+ T cells were recovered during acute infection. Many of the T-cells were low-affinity for the infecting virus, but were cross-reactive with higher affinity towards other strains, displaying aberrant responses with more cytokine production and less degranulation [105].

Despite the evidence on T-cell dependent immunopathology, virus-specific T-cell responses have also been reported to provide protection. Prior exposure to DENV can trigger more rapid T cell responses to ZIKV infection [103]. In mice too, cross reactive CD8 + T cells have been reported to provide protection against DENV and ZIKV [107]. It is however worth noting that most animal studies on T-cells have been performed with interferon-deficient mice, which do not accurately reflect the disease phenomenon seen in infected patients.

#### Antibody responses to flaviviruses

Neutralising antibodies generated as a consequence of infection or vaccination are critical for developing immunity to viral infections. Among flaviviruses, successful vaccines exist for YFV and JEV. Vaccine development against other flaviviruses such as DENV and ZIKV has been partly confounded because of high sequence conservation and immunological cross-reactivity among their envelope glycoproteins. Antibody responses to these viruses are primarily elicited to the E, prM and NS1 proteins [108,109]. The E-protein is the primary target of neutralising antibodies and comprises three domains – EDI, containing the N-terminus, EDII has the fusion loop that mediates endosomal fusion and EDIII is a highly variable immunoglobulin like domain that is required for attachment to host cells. All three structural domains of E have neutralising epitopes; however, antibodies to EDIII appear to be the most potent [110]. A majority of monoclonal antibodies isolated from ZIKV infected patients display antibodies to EDI/II domains and cross-react with those of DENV on account of the high sequence homology of these epitopes. In contrast anti-EDIII antibodies are a minor component of the humoral response and are specific to DENV or ZIKV [111,112].

Antibodies to prM are also produced upon DENV – and WNV-infection. In a study designed to test the neutralising potential of anti-DENV antibodies, B cells isolated from infected individuals were used to produce antibodies. Anti-prM antibodies dominated the B cell response, most of which cross-reacted with all four DENV serotypes [96]. They did not neutralize, but instead were found to enhance infection [96].

Besides antibodies to E and prM, those against NS1 are abundant in patient serum [113]. The virus NS1 protein is multifunctional and exists in both monomeric and oligomeric forms. The monomeric version participates in ER remodelling during replication; the dimers have been found associated with membranes of infected cells, whereas high levels of the hexameric form is secreted out are found in patient sera during acute infection [114].

#### Antibody-dependent enhancement in flavivirus infections

**Figure 3.**
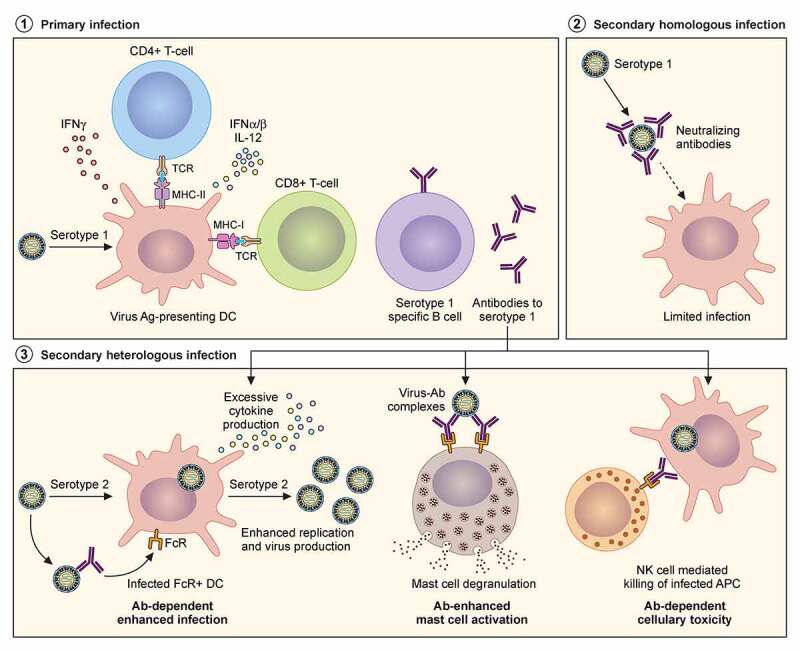
**Adaptive immune responses to homologous and heterologous flavivirus infections**. Severity of disease outcomes in dengue and Zika infection can be driven by pre-existing immunity against one of them. Primary infection results in production of high affinity neutralising antibodies against the infecting strain. When followed by a secondary infection with the same virus as that of the primary infection, existing memory B and T cells may lead to effective neutralisation of the virus, thereby providing protection. However, in the event of a secondary infection with a heterologous virus (either a different serotype of DENV or with ZIKV), cross-reactive antibodies at sub-neutralising concentrations result in binding and opsonization of the infecting viral particles. These antibody-virus complexes can be taken up by FcR-expressing cells such as monocytes, macrophages and mast cells, causing increased viral replication and hyperinflammatory responses. The pre-existing antibodies can also bind to infected cell surfaces causing NK cell mediated lysis via antibody-dependent cellular cytotoxicity

Antibody-dependent enhancement (ADE) describes the phenomenon of increased disease severity seen in subsequent DENV infections in patients with prior exposure, as well as in primary infections in infants born to DENV-exposed mothers. A primary infection elicits neutralizing responses against the infecting strain. After a secondary infection with a homologous virus, an individual therefore remains protected; however, with a heterologous virus containing structural proteins differing by 30–35% in sequence, cross-reactive antibodies to the primary virus may not be present at either sufficient concentration or of avidity required to neutralize the second virus. These non-neutralizing antibodies bind and opsonize viral particles, which facilitate entry into FcR-expressing myeloid cells, resulting in higher viral loads [115,116] (Figure 3). ADE can be observed both *in vitro* and *in vivo* by low affinity antibodies or those present at sub-neutralizing concentrations. Apart from exacerbating the viral load, they also have potential to increase infection in cells that are typically resistant to flaviviruses, such as mast cells. Degranulation of mast cells was found to increase upon antibody sensitization in vitro, particularly with cross-reactive antibodies followed by viral challenge with different serotypes [117]. Evidence from a paediatric cohort has shown that low antibody titres from prior DENV infection is correlated with an increased risk of severe disease, underscoring the propensity of cross-reactivity and poor neutralising properties of pre-existing antibodies to cause ADE [118]. In the same vein, increased hospitalization from natural DENV infection in children immunized with the tetravalent live attenuated vaccine produced by Sanofi, compared with control vaccines was believed to be the result of ADE from antibodies generated against the vaccine strain [119]. Finally, multiple *in vitro* and *in vivo* studies have shown that antibodies to DENV cross-react with ZIKV to drive ADE [120,121] and vice-versa [122]. Interestingly, pre-existing immunity to DENV was found to have minimal impact on the innate immune responses to Zika infection as measured by transcriptomic and cytokine profile analyses in a paediatric cohort [123].

Several gaps exist in our understanding of flavivirus interaction with that of the host immune system; the most imperative is to determine what drives immunopathology in heterologous flavivirus infections and the contribution of the different branches of host immunity to this phenomenon. This will become possible in future studies with the advent of better *in vitro* and *in vivo* models (described in section 4 (Experimental models)) as well as mass spectrometry-based approaches (section 5) to determine alteration of immune responses at the proteome level.

## Clinical features of flavivirus infections

Flavivirus infections are often accompanied by variable and often unpredictable clinical manifestations, disease severity and long-term persistence. Haemorrhagic fever, vascular permeability, encephalitis, biphasic fever, flaccid paralysis, and jaundice are typical manifestations. The disease phenomena can be broadly classified into two categories – one exemplified by DENV, which includes systemic infection, haemorrhagic fever and endothelial leakage, while the other by ZIKV and WNV, leading to neurological complications.

Both DENV and ZIKV infections can display a variety of clinical presentations ranging from asymptomatic infection to an influenza-like viral illness that appears similar in its early stages to illnesses caused by infections with other arboviruses, such as Chikungunya[124]. Typical signs of infection are rash, conjunctivitis, headache and fatigue [125], but are generally considered to be self-limiting[126]. Although patients infected with ZIKV in general are less likely to be febrile during presentation, a proportion of individuals develop a clinically apparent febrile illness, which resolves within a few days [127,128]. However, during the outbreak of 2015, ZIKV infection often developed into severe disease, resulting in multi-organ failure [129,130].

Apart from the typical symptoms, ZIKV infections are associated with complications, such as neurological disorders and Guillain Barré Syndrome (GBS) reported during the ZIKV epidemic in French Polynesia [131,132]. Notwithstanding these reports, ZIKV is considered less neuro-invasive in adults compared to other encephalitic flaviviruses (e.g., WNV and TBEV), with limited incidents of meningitis and encephalitis[133]. ZIKV is able to target neural progenitor cells[134], which may explain the associated neurodevelopmental defects. ZIKV has also been reported to infect the eye, causing uveitis in adults [135,136] and conjunctivitis in up to 15% of patients [127,136,137].

One of the disease complications of ZIKV infection during the last outbreak in North America was its association with microcephaly and fetal abnormalities. Approximately 30% of foetuses from ZIKV-infected pregnant women in Brazil revealed some form of irregularity [138,139]. The presentation of congenital ZIKV infection also included cerebral calcifications, microcephaly, intrauterine growth restriction, and fetal demise. In French Polynesia, increased risk of microcephaly was associated with ZIKV infection, with 95 cases occurring per 10,000 women infected in the first trimester[140]. A retrospective study of 23 children with diagnosis of congenital infection demonstrated calcification, malformation of cortical development, decreased brain volume, and hypoplasia of the cerebellum or brainstem[141]. Loss in hearing and eyesight were also reported for congenital ZIKV infection[142]. A further complication was its association with Guillain-Barré syndrome reported in several countries [143,144].

In line with ZIKV symptoms, DENV infection can also produce a wide spectrum of clinical presentations, as well as be asymptomatic. While symptomatic cases can be self-limiting, a proportion of cases progress to a severe disease form, characterized by systemic vascular leakage, multi-organ failure, and profound shock [145,146]. The clinical presentation of infection with DENV is categorised as mild versus severe disease as per the revised WHO classification and differentiated into febrile, critical and recovery phases[147]. Various forms of cardiac complications are also known to arise during dengue infections, resulting in inclusion of myocarditis in the revised definition of severe dengue; however, due to a lack of screening in dengue-endemic countries the true incidence of myocarditis remains unknown.

## Experimental models to study flavivirus pathogenesis

### In vitro models

Both DENV and ZIKV display broad tissue tropism, which includes lymph nodes, spleen, liver and bone marrow. The primary target cells for DENV are immune cells; in particular, dendritic cells (DCs), monocytes, macrophages are infected, but also lymphocytes and endothelial cells become infected during the course of disease. Many primary cell cultures, including epithelial, endothelial and fibroblasts have been shown to support viral replication *in vitro*. Infection of human peripheral blood mononuclear cells have demonstrated that CD14+ cells, but not CD3+ or CD19+ cells are permissive to infection. A range of human cell lines are now used for propagation of virus strains, diagnostic assays, antiviral screens and host-virus interaction studies [148,149]. More sophisticated *in vitro* cultures for modelling neurological manifestations of DENV, such as polarized monolayer brain endothelial cells and their co-cultures with astrocytes have also been developed. One of the caveats of the *in vitro* studies is the use of laboratory-adapted virus strains, which infect a broad range of cell lines, grow to high titres and form plaques, but may not reflect physiological settings and differ from results obtained with low-passage isolates. Dominant mutations in the laboratory-adapted strains are often acquired in cell culture and confer phenotypes that are not physiologically relevant. In fact DENV isolates have been reported to vary in their capacity to infect the same cell type *in vitro*, depending on the virus genotype and passage history[150].

### In vivo models

Appropriate animal models for flavivirus infection are lacking. However, a range of murine models have been developed to study specific aspects of infection biology. Two different approaches have been taken: to induce human-like disease in immunocompetent mice and to mimic human disease in immunodeficient mice that are engrafted with human cells. None of them recreate the full spectrum of dengue disease.

In humans, both DENV and ZIKV viruses antagonize the type I interferon (IFN) response, via its NS5 protein (described in section 2.1.5), which promotes proteasomal degradation of STAT2 [85,151], and facilitates infection. In contrast, ZIKV or DENV NS5 does not bind to or degrade mouse Stat2, which in part, explains why immunocompetent mice are not susceptible to these viruses. To develop models of dengue and Zika infections in mice, immunocompetent animals have been intravenously inoculated with high titres of ZIKV[152] to circumvent innate immune responses. Most common however, is to subcutaneously inoculate *Ifnar1^−/−^* or *Ifnar1*^−/−^ *Ifngr*^−/−^ double knock-out mice (AG129) [153,154]. More recently an immunocompetent mouse model carrying a knock-in of human STAT2 has been developed for Zika infection [155,156].

To circumvent flavivirus resistance of adult mice, more susceptible neonatal mice have also been used. Wild-type newborn mice infected with a ZIKV isolate from Puerto Rico displayed severe pathologies, such as neurological malfunction, weight loss, and death[157]. In contrast to the Ifnar^−/ –^ mice, which showed infiltration by neutrophils and macrophages, the neonatal wild-type mice displayed CD8+ T cell infiltration into the central nervous system, in line with other models of neuro-invasive flaviviruses.

In vivo experiments in immunocompromised mouse models have shown that ZIKV accumulates in lymph nodes, bone marrow, spleen, brain, spinal cord, and eyes, recapitulating features of disease progression in humans. Neurological presentation in mice, however, does not represent neurological defects of Guillain-Barré syndrome but instead arises from cytotoxicity of neural progenitor cells. *Ifnar1^−/−^* mice also display high viral load in their testes. In a separate study, 6-week-old mice deficient in IRF3, IRF5, and IRF7 were seen to be more susceptible to a Cambodian clinical isolate of ZIKV and developed CNS infection that resulted in cytotoxicity of neurons [158].

To model congenital defects, experiments have been performed in pregnant female mice. A ZIKV variant from French Polynesia could infect different trophoblasts and placental endothelial cells, and also able to cross the placenta into the fetal brain [159]. Immunocompromised *Ifnar1*^−/−^ mice infected subcutaneously with ZIKV also developed similar pathologies [159,160]. In contrast, when wild-type C57BL/6 mice were inoculated intravenously with a Brazilian variant of ZIKV, the fetuses remained uninfected unless inoculated with unusually high doses[152]. However, subcutaneous inoculation of pregnant *Ifnar1^−/−^* mice with a Brazilian clinical ZIKV isolate led to reduced fetus sizes[161]. These disease phenotypes have been reported in humans, including complications of intrauterine growth, microcephaly, and fetal demise [138,162].

Non-human primate (NHP) models have also been used to study ZIKV and DENV infection specifically in relation to virus tropism, pathogenesis and for development of drugs and vaccines. Vertical transmission of ZIKV virus through blood barrier to the foetus has been observed in rhesus macaque models. A recent study has also shown NHPs could act as reservoir to propagate mosquito-borne enzootic transmission of ZIKV and DENV [163,164]. High throughput serological screening tools have revealed the presence of antibodies against these viruses in NHPs[165]. Viremia and cellular immune responses in primates sequentially infected with different DENV serotypes demonstrated cross-reactivity of T-cells upon infection with higher responses directed towards the primary infecting serotype, in line with human infections. In particular, a 100aa region of NS2A protein of DENV was reported to induce T-cell response in macaques[166]. DENV infection also induced responses in central memory CD4^+^ or CD8^+^ cells and NKT cells in a marmoset model[167]. Another study analysed cellular immune response elicited against NS1, NS3 and NS5 proteins of DENV during primary infection in Indian rhesus macaques, to identify immunogenic regions in these proteins that may activate CD4^+^ cytotoxic lymphocytes which could be useful for vaccine trials[168].

Brain organoids from NHP (chimpanzee) pluripotent stem cells showed that Brazilian ZIKV did not replicate or induce cell death in them, while ZIKV derived from zoonotic agent associated with primates in Africa were adapted to these cells[169]. Unlike findings from human patients, prior infection with heterologous flaviviruses – DENV2, DENV4 or YFV did not alter ZIKV titres and immune response in rhesus macaques[170]. A detailed review of ZIKV and DENV pathogenesis in NHP can be found elsewhere [171–173].

## Mass spectrometry-based approaches to study flavivirus infection biology

Mass spectrometry (MS)-based proteomics has become a cornerstone technology for studying different dimensions of the proteome, such as abundance levels, protein–protein interactions, post-translational modifications (PTMs), subcellular localization of proteins, and protein synthesis and degradation rates[174]. The combined effect of these different dimensions plays an integral role in mounting protective immune responses during infection and enables a return to homeostasis after clearing the infection. Understanding key events in the infection process can lead to better diagnosis and/or treatment strategies for flaviviral infections. Below we highlight MS-based proteomics approaches that have been used to study flavivirus infection biology.

### Flavivirus-host protein–protein interactions

#### Virus-host receptor interactions

The first key step in the flavivirus life cycle is the attachment of viral glycoproteins to the host cell surface, followed by interaction with one or several host cell surface receptors. Flavivirus-host cell surface receptor interactions subsequently trigger virion internalisation by endocytosis [175]. Even though attachment and entry are key steps in the viral life cycle, host cell surface receptors required for flavivirus entry remain ill-defined. MS-based proteomics has been used to identify candidate flavivirus receptors on host cells. For example, virus overlay protein binding assays and MS-based proteomics have been used to identify surface-localised vimentin as interacting partner of DENV2 EDIII of the receptor binding domain on human vascular endothelial cells [176]. Affinity purification in combination with MS-based proteomic analysis (AP-MS) has been used to identify glucose-regulated protein 78 (GRP78) as an interacting partner of a recombinant JEV EDIII in plasma membrane fractions from mouse neuronal (Neuro2a) cells [177]. Neutralization assays using anti-GRP78 antibodies and small interfering RNA (siRNA)-based gene silencing prior to JEV infection revealed a role for GRP78 in viral entry in primary mouse cortical neurons, Neuro2a cells, and human epithelial Huh-7 cells. Interestingly, GRP78 was also shown to have post-virus entry inhibitory effects on viral replication and protein synthesis. Using a similar approach, plasmalemma vesicle-associated protein (PLVAP) and gastrokine3 (GKN3) were identified as interacting partners of the JEV envelope protein in plasma membrane fractions of BALB/c mouse brains [178]. PLVAP and GKN3 were shown to be membrane receptors that govern JEV entry and propagation in murine neurons. A global ZIKV-human AP-MS screen identified ATPase Na^+^/K^+^ transporting subunit alpha 1 (ATP1A1) as an interacting partner of ZIKV non-structural protein NS4B [179]. ATP1A1 has previously been identified as an anti-ZIKV target during an antiviral screen of FDA-approved drugs [180]. However, it remains to be determined whether ATP1A1 is indeed a ZIKV attachment or entry receptor. The above-mentioned studies all relied on single affinity tagged viral proteins as bait for the identification of host cell surface flavivirus receptors. Several recent studies have, however, exploited intact viral particles as bait for flavivirus receptor identification.

MS-based proteomic analysis in combination with virus overlay protein binding assays identified annexin II as a DENV2 interacting protein on Vero cells [181]. Neutralization assays revealed reduced viral infectivity when using an anti-annexin II antibody before DENV infection. Protein correlation profiling has been used to identify HSP70 in fractions co-migrating with ZIKV particles when separating intact protein complexes by density-gradient centrifugation and size-exclusion chromatography [182]. HSP70 is part of a family of conserved heat shock protein chaperones that are ubiquitously expressed on cells and have been shown to play a role in hepatitis C virus (HCV), DENV, and JEV infection [183–185]. More recently, using an elegant chemical proteomics strategy Srivastava et al. conjugated a multifunctional chemical probe to the ZIKV surface to covalently crosslink host interactors [186]. Importantly no effect on ZIKV infectivity was observed after labelling with the chemical probe, allowing for near native-state interactions to be captured. In total, 500 proteins were identified with several of these previously implicated in viral infection. In particular, neural cell adhesion molecule (NCAM1) was identified as ZIKV receptor and neutralization assays confirmed a role for NCAM1 in ZIKV binding and entry in human U-251 MG and Vero cells. NCAM1, a cell adhesion glycoprotein of the immunoglobulin (Ig) superfamily, has previously been identified as a receptor for Rabies Virus, indicating that this could be a receptor targeted by several viruses [187].

Even though MS-based proteomics has played an integral role in our understanding of flavivirus-host cell surface receptor interactions, large knowledge gaps still exist regarding the host receptor repertoire that flaviviruses exploit for viral attachment and entry. Advances in chemical proteomics and crosslinking mass spectrometry workflows will facilitate further characterisation of flavivirus receptors using intact viral particles.

#### Flavivirus-host protein–protein interactions

Once internalised, flaviviruses hijack the host cell machinery to enable viral replication and propagation. Viral exploitation of the host cell machinery and immune evasion is achieved through several mechanisms, one of which includes direct viral-host protein–protein interactions (PPI). AP-MS is by far the most commonly used strategy for studying viral-host protein–protein interactions and has been used in dedicated studies to identify interactors of single flaviviral bait proteins. We refer the reader to excellent reviews that cover these studies [175,188,189]. Here we highlight large-scale AP-MS efforts that identified global flavivirus-host protein–protein networks. Pioneering genome-wide flavivirus-host PPI studies have focussed on ZIKV, DENV, and WNV. To gain insight into host pathways involved in ZIKV neuropathogenesis, Scaturro et al. expressed epitope-tagged ZIKV proteins in human SK-N-BE2 neuroblastoma cells and used AP-MS analysis to identify viral protein interactors [179]. Using this approach, 386 ZIKV interacting proteins were identified from 484 high-confident interactions. ZIKV interacting partners could be linked to neuronal development and neurological diseases, such as CLN6, BSG, CEND1, RBFOX2, CHP1, and TMEM41, which all interacted with ZIKV NS4B. Furthermore, ZIKV capsid was shown to interact with LARP7, LYAR, and NGDN, which are also linked to neuronal development and neurological diseases. Ectopic expression of ZIKV-NS4B during differentiation of human neural progenitor cells (hNPCs), derived from induced pluripotent stem cells, reduced the expression of proteins involved in neuronal differentiation, including some implicated in neurological diseases. Importantly, similar effects were observed in hNPCs infected with ZIKV. These results indicate possible molecular mechanisms of ZIKV neuropathogenesis, which could lead to microcephaly. A combined AP-MS and proximity labelling approach has been used to map the ZIKV-human interaction network [190]. Proximity labelling of interacting partners is achieved by fusing an engineered biotin labelling enzyme to a bait protein of interest, followed by ectopic expression in a host cell [191]. Using this combined approach, Coyaud et al. identified 1,224 ZIKV interacting proteins from 3,033 high confidence interactions of 10 ZIKV proteins expressed in HEK293T cells. This analysis revealed extensive targeting of ZIKV proteins to diverse host organelles that can impact several biological functions. For example, the authors suggested that ZIKV proteins can manipulate host cell membrane remodelling (ZIKV C), Cajal Body formation (ZIKV NS5), and lysosomal (NS2B and NS3) and peroxisomal functions (NS2A). To identify pan-flavivirus and flavivirus-specific host interactions, Shah et al. generated DENV-host and ZIKV-host PPI networks in both human and mosquito cells using a combined AP-MS and RNAi screening approach [192]. Initially, a DENV-human PPI network was generated using AP-MS analysis of epitope-tagged DENV2 proteins expressed in human HEK293T cells. In total, 198 high-confidence DENV-human PPIs were identified, which consisted of known DENV-human interactions that were enriched in biological processes consistent with DENV bait localisation in the host cell. Comparison of DENV-human PPI and ZIKV-human PPI networks revealed 28 shared flavivirus-host PPIs. Several NS5 interactors were shared between DENV and ZIKV, with multiple subunits of PAF1C identified as interactors of both viruses. PAF1C is a chromatin-associated complex required for transcriptional elongation, which was shown to dampen expression of interferon stimulated genes in a NS5-dependant manner. Furthermore, the authors generated a DENV-mosquito PPI network using *Aedes aegypti* Aag2 cells to identify shared PPIs and pathways between both human and mosquito hosts, identifying key processes that could serve as pan-flavivirus antiviral targets. For example, pharmacological inhibition of SEC61 decreased replication of both viruses in both hosts. Additionally, ZIKV but not DENV NS4A expression induced microcephaly in an ANKLE2-dependent manner. The ZIKV NS4A-ANKLE2 interaction therefore reveals a possible mechanism of ZIKV-mediated neuropathogenesis. Finally, to generate a WNV-human PPI network, Li et al. expressed affinity-tagged WNV proteins in HEK293 cells followed by AP-MS analysis [193]. The authors identified 259 WNV-interacting proteins, and RNAi screening revealed 26 proteins that interacted with WNV proteins to influence infection. Comparative analysis highlighted several capsid-interacting host proteins shared across flaviviruses, including DENV, ZIKV, and WNV, even though protein sequence similarity of the capsid proteins were low. This indicates conservation of structural and functional properties in the viral capsid that drive interactions with host proteins. In particular, WNV capsid was shown to interact with PYM1, a protein involved in RNA processes such as exon-junction complex and nonsense-mediated decay as a way of subverting antiviral responses.

Taken together, these large-scale PPI network maps reveal a systems-level overview of flavivirus infection biology. When combined with other multidimensional data and analysed in a system biology context [194], these network maps can reveal key aspects of flavivirus infection biology that could be exploited in pan-flaviviral or flaviviral-specific antiviral therapies.

#### Flavivirus RNA-host protein interactions

Flaviviruses not only exploit viral proteins to interact with and manipulate host responses, they can also sequester host nucleic acid-binding proteins using genomic or subgenomic RNA. Early pioneering studies used RNA affinity chromatography to identify host proteins that bind to flaviviral RNA, including 3ʹ untranslated regions (UTRs), and subgenomic RNA. Several groups identified flavivirus RNA-binding host proteins such as TRIM25, DDX6, G3BP1, G3BP2, Caprin1, USP10, FMRP, FXR1, FXR2, QKI, and ERI3 in whole-cell lysates using this approach [195–200]. Recent methods developed to capture *in vivo* flaviviral RNA-host protein interactions using RNA-protein crosslinking allowed identification of DENV RNA interactors in Huh7 hepatoma cells [201]. Following infection, DENV RNA-host protein complexes were crosslinked with UV light (254 nm), and isolated by affinity purification using biotinylated antisense DNA oligonucleotides complementary to DENV RNA. In total, 12 host proteins were identified as high-confidence flaviviral RNA-binding proteins. Importantly, siRNA-mediated silencing of CSDE1, YBX1, HNRNPC, and PABPC1 caused a reduction in viral titres, while silencing of NCL increased viral titres. Using a modified thiouracil cross-linking mass-spectrometry approach (TUX-MS), originally developed to identify human proteins that bind to poliovirus RNA[202], Viktorovskaya et al. identified DENV RNA-binding proteins in Huh7 cells[203]. In this approach, 4-thiouridine is incorporated into RNA by supplementation in the growth media, followed by conversion of 4-thiouridine to UMP by a uracil phosphoribosyltransferase expressed in the host cells. UMP is subsequently converted to thiouridine triphosphate by cellular kinases, which serves as a zero-distance cross-linker upon exposure to ultraviolet (UV) light. Proteins that were bound to RNA were cross-linked by UV exposure, and DENV RNA-host protein complexes were isolated using DENV antisense biotin-labelled antisense DNA oligonucleotides that recognise viral RNA. Using metabolic labelling with TUX-MS, the authors were able to identify several viral and host proteins known to associate with DENV genomic RNA, as well as 79 proteins not previously linked with DENV infection. Notably, siRNA knockdown of NONO, HMCES, RBMX, hnRNP M, and hnRNP F reduced DENV titres. To identify pan-flaviviral RNA-associated human proteins, Ooi et al. used the ChIRP-MS (Comprehensive Identification of RNA-binding Proteins by Mass Spectrometry) workflow [204] to identify proteins that are associated with DENV and ZIKV RNA in Huh7.5.1 cells [205]. Briefly, infected cells were crosslinked with formaldehyde, and flaviviral RNA-protein complexes were isolated using biotinylated oligonucleotides that that bind DENV and ZIKV RNA, respectively. Flaviviral NS3 and NS5 proteins were highly enriched in the ChIRP-MS data, while several other viral proteins were also detected. Furthermore, 464 human proteins were identified that were associated with flaviviral RNA, such as MOV1011, YBX112, ADAR13, and SND1 with several that were independently identified in genome-wide CRISPR/Cas9 knockout screens. Among these were ER-associated proteins such as vigilin and RRBP1, which had not previously been linked to flavivirus infection. Huh7.5.1 cells lacking RBP1 had reduced viral RNA loads for DENV, and ZIKV, while Huh7.5.1 cells lacking vigilin had reduced viral RNA loads for DENV, ZIKV and Powassan virus (POWV), another member of the flavivirus genus. Interestingly, this effect was not observed for Chikungunya virus. Furthermore, RRBP1 and vigilin was shown to bind DENV and ZIKV RNA to impact viral RNA stability, replication, and translation. These results indicate that vigilin and RRBP1 are pan-flaviviral RNA-binding host proteins that are exploited during infection. Together these studies highlight diverse host proteins that can bind flaviviral RNA to facilitate viral replication.

#### Global alterations in posttranslational modifications during flaviviral infection

Post-translational modifications (PTMs) play important regulatory roles in biological processes by influencing protein function, stability, and localisation. PTMs impart these pleiotropic effects by increasing the overall chemical diversity of the proteome through covalent attachment of chemical groups (phosphorylation, acetylation) or small proteins (ubiquitylation and ubiquitin-like modifiers) to defined residues on substrates. Given the importance of PTMs in host cell signalling and proteome dynamics, it is not surprising that flaviviruses exploit PTMs to alter host responses during infection.

Phosphorylation is a well-characterised PTM critical for signal transduction. Zhang et al. used label-free quantitative phosphoproteomics to identify altered protein phosphorylation during WNV infection in human glioblastoma (U251) cells[206]. Differentially regulated phosphopeptides were involved in the regulation of gene expression, RNA processing, RNA splicing, signal transduction, and apoptosis. Enrichment analysis revealed overrepresentation of c-Jun N-terminal kinase 1/mitogen-activated protein kinase (MAPK) motifs, and that several phosphoproteins were linked to inflammatory responses. Phosphorylated targets (GSK3B, PNKP, and RB1) correlated with altered NF-κB signaling and increased inflammatory cytokine production during WNV infection. Using a similar phosphoproteomics approach, Ye et al. showed that JEV infection in U251 cells is also associated with increased phosphorylation of proteins involved in inflammatory responses [207]. Pathway analysis of regulated phosphoproteins revealed several kinase-mediated signaling pathways (AKT1, PRKACA, and JNK1) being altered during JEV infection, with JNK1 (MAPK8) substrates significantly overrepresented during infection. Pharmacological inhibition of the JNK pathway reduced inflammatory responses in the brains of JEV-infected mice, with a concomitant increase in survival of infected animals. These results suggest that JNK signaling is important for JEV-induced encephalitis and can serve as potential therapeutic target. Similarly, phosphoproteomic analysis of ZIKV-infected SK-N-BE2 cells revealed altered ATM, AKT-mTOR, and ERK-MAPK signalling pathways [179]. Interestingly, proteins (p38 MAPK, MARCKS, and DPYSL2) involved in neurite outgrowth and brain development were also shown to be dephosphorylated during ZIKV infection. These results highlight some of the signalling pathways that ZIKV exploit to induce autophagy, decrease cell proliferation, and impair neurogenesis during infection.

Ubiquitylation is an elaborate post-translational modification that is formed by the covalent attachment of the 76-amino acid protein ubiquitin to substrates. Interestingly, ubiquitin itself can be further ubiquitylated on any of its seven lysine residues (K6, K11, K27, K29, K33, K48, and K63) and the N-terminal methionine (M1) to form complex ubiquitin chain architectures. These diverse chain architectures have defined cellular functions. Recently our group performed a proteomic screen to identify monoubiquitylated substrates in DENV-infected human liver cancer Hep2G cells [208]. Using a modified HA-Ub-L73P probe, where all lysines were mutated to arginines to prevent polyubiquitylation (HA-Ub-L73P*), we identified several lipid droplet-associated proteins that were differentially monoubiquitylated during DENV infection. AUP1, a lipid droplet-localized type-III membrane protein, was shown to have reduced levels of monoubiquitylation during DENV infection and to interact with DENV NS4A. This interaction exploited the acyltransferase activity of AUP1 to alter lipophagy and promote viral production, which seems to be a pan-flavivirus mechanism.

#### Global proteome remodelling during flaviviral infection

Remodelling of host and viral PPIs, nucleic-acid interactions, and host cell signalling during infection will ultimately impact the host proteome for sustained viral replication and propagation. To identify proteome alterations during flavivirus infection, quantitative proteomic analysis on WNV-infected Vero cells has revealed several differentially regulated proteins linked to transcription and translation, cell mobility, chaperones, metabolism, apoptosis, the ubiquitin-proteasome pathway, nucleotide metabolism, and protein transport [209]. Similarly, DENV infection in K562 cells revealed altered macromolecule biosynthetic processes, RNA splicing, vesicle transport, and membrane organization [210]. Altered phosphoproteins identified in the same study were also linked to nucleic acid-associated processes including negative regulation of nucleobase, nucleoside, nucleotide, and nucleic acid metabolic process, negative regulation of gene expression, chromatin modification, RNA splicing, cell cycle, mRNA processing, and regulation of transcription. Not surprisingly, DENV infection has also been shown to cause upregulation of proteins involved in the ubiquitin-proteasome system [203,211], upregulation of the type-I interferon-response[212], and decreased expression of proteins involved in central metabolism[213]. Furthermore, proteome analysis of JEV-infected hNS1 cells revealed a strong ER unfolded protein stress response and alterations in apoptosis, with upregulation of GRP78, calreticulin, PHB, hnRNPC, and Hyou1. Importantly, upregulation of GRP78, PHB, and hnRNPC was shown in hNPCs isolated from human foetus, and increased mRNA levels could be detected in post-mortem samples from human encephalitis patients[214]. Similarly, JEV-infected mouse brain and mouse Neuro2a cells also showed differentially expressed proteins involved in the ER stress response (HSP90B1, HSPA5, HSPA8, CALR, ATP5B, IDH3A), as well as protein transport (VIM, ACTG1, PRDX4, PDIA6), metabolism (PDHB, PRDX6, LDHB, ALDOC, YWHAG, GAPDH, PRDX6, PDXP, CKB, ENO2) and apoptosis (HSP90B1, HSPA5, HSPA8, CALR, ATP5B, IDH3A) [215]. Proteomic analysis of viral replication organelle-enriched fractions in JEV-infected cells identified components of the ESCRT complex (CHMP4B and ALIX) [216]. The ESCRT complex was shown to be important for late-stage infection and required for viral particle formation on the ER membrane of both JEV and DENV. Taken together, these studies highlight marked changes in the host proteome during flaviviral infection that are driven by altered nucleic acid-associated processes, host immune responses, and host metabolism. Furthermore, the importance of ER membrane remodelling and ER stress during flaviviral infection is highlighted.

To gain proteome-level insights into ZIKV-induced changes in neuronal development, Scaturro et al. analysed the proteomes of ZIKV-infected hNPCs during differentiation into neurons [179]. ZIKV-infection led to a robust upregulation of type-I interferon-stimulated genes (such as OAS3, TRIM25, ISG15), while several proteins important for neuronal differentiation were downregulated. Similarly, ZIKV-infection of primary human neural progenitor cells affected processes such as neurogenesis, neural differentiation, and neuron projection development [217]. DCX, a protein involved in neuronal migration and development, was shown to be downregulated in primary human fetal neural progenitor cells as well as fetal mouse brains during ZIKV infection [218]. These results indicate that ZIKV infection alters several proteins linked to neuronal development and differentiation, which could play a role in ZIKV-induced neuropathogenesis. Interestingly, proteomic analysis of ZIKV-infected *Aedes albopictus* C6/36 cells revealed the ubiquitin-proteasome system, ER unfolded protein response, immune response, and metabolic processes as regulated during infection, which is in line with human responses to infection. Comparison of different host responses to infection can identify pan-flavivirus targets for antiviral strategies aimed at controlling ZIKV spread, or can serve as targets for treatment options during infection.

### Clinical flavivirus proteomics

MS-based proteomics has become a powerful technology in translational infectious disease research. Clinical flavivirus proteomics studies have mainly focussed on biomarker discovery in plasma or serum of infected individuals since blood is an easily obtainable clinical specimen that requires minimally invasive procedures for collection. Analysis of the serum proteome of patients with dengue fever (DF), malaria, and healthy subjects (HS) revealed 48 proteins that were differentially expressed between DF patients and HS, with 26 proteins identified by MALDI-TOF/TOF [219]. The majority of these proteins were well-known highly abundant serum and plasma proteins, such as apolipoproteins, complement, α1-antichymotrypsin, serum amyloid P, and haptoglobin. Interestingly, 11 candidate proteins were differentially expressed between DF and malaria patients, with differences in clusterin, hemopexin, and haptoglobin levels confirmed by western blot. Upregulation of apolipoproteins (APO A‐1) in DENV-infected patients has subsequently been shown in an independent study [220]. Proteomic analysis of virion-enriched fractions purified from plasma of DF patients and patients with severe dengue (SD) led to the identification of DENV envelope protein as well as 188 host proteins in the virion-enriched fractions [221]. Unsurprisingly, several highly abundant serum and plasma proteins involved in the coagulation cascade, complement pathway, and acute phase response were identified. Differences in OLFM4 and PF4 were validated in an independent cohort using ELISA, and indicates that these two proteins could have prognostic value. To identify correlative markers of disease severity, Brasier et al. analysed the plasma proteomes of DF, dengue fever complicated (DFC), and severe dengue haemorrhagic fever (DHF) patients [222]. Two hundred and twenty-one proteins were differentially regulated between DF and DHF patients, with acute phase and coagulation cascade pathways enriched in DHF patients. Selected reaction monitoring (SRM), a targeted proteomics approach, was used to verify 15 differentially expressed proteins, including DENV NS1, CFD, A1AT, A2M, CO4A, TPM4, FRIL, DESP, AG2L, KRT1, IGJ, and HPT. These 15 proteins, in combination with clinical variables, could accurately predict DF, DHF, and DFC disease using a random forest classifier. Several highly abundant serum proteins, such as alpha-2 macroglobulin, angiotensinogen, apolipoprotein B-100, serotransferrin, and ceruloplasmin, have independently been shown to be upregulated in DHF cases compared to HS [223]. Furthermore, angiotensinogen and antithrombin III expression levels have been shown to correlate with dengue with severe plasma leakage in paediatric patients [224]. The majority of clinical proteomics studies to date have focussed on DENV disease progression. More recently unique protein signatures in patient serum were identified that could distinguish between ZIKV and DENV infection[225]. Thirteen differentially expressed proteins were identified, with 10 upregulated in ZIKV compared to DENV infection. CA2 protein levels were shown to be upregulated in ZIKV compared to DENV-infected patient samples in an independent cohort. Furthermore, three proteins (FGA, PF4V1, and PPBP) were shown to have high predictive value in distinguishing between ZIKV and DENV infection.

These flavivirus clinical proteomic studies have revealed key host responses to infection and have paved the way forward for future studies. Since the majority of biomarker candidates identified to date comprise highly abundant serum or plasma proteins and inflammation-related markers, it will be difficult to implement as diagnostics as specificity is lacking, such as a virus-specific peptide. However, these markers can serve as candidates for disease progression or treatment response monitoring in infected individuals. Ultrafast proteomic methods that enable scaling up sample analyses per day [226] can generate data sets that are ideal for machine learning-based approaches to improve biomarker discovery. It is envisioned that such approaches will be integral to flavivirus clinical proteomics in the future.

## Genome-wide screening approaches to identify host-flavivirus interactions

Apart from proteomics-based approaches described above, functional genomic screens have been also revealed host-flaviviral interactions on a genome-wide scale. RNA interference (RNAi) and Clustered Regularly Interspaced Short Palindromic Repeats (CRISPR)/Cas9 screens have been used in either loss-of-function or gain-of-function screens in DENV [227–230], ZIKV [227], WNV [231–234], HCV [229], and YFV [235] infections to identify host factors important for flaviviral pathogenesis. Many of the key findings from these screens have been summarised elsewhere [236,237]. These functional genomic screens identified several host processes important for infection, including entry factors [227], endocytosis [231], vesicle trafficking [228,231] and immunity [231,235]. A major overlap in the above-mentioned studies with the proteomics-based approaches are ER-associated complexes, which include the ER membrane protein complex (EMC), oligosaccharyl-transferase (OST) complex, ER-associated signal peptidase complex (SPCS), translocon-associated protein (TRAP), complex and the endoplasmic reticulum-associated degradation (ERAD) pathway. Many of these findings have been independently corroborated in genome wide RNAi and CRISPR studies. For instance, ERAD components (HRD1, DERL2, UBE2J1/UBC6, UBE3A, SEC61G, SEC61A1, UFD1L and NSFL1C) were identified as dependency factors for WNV and DENV infection [231]. Additionally, RNAi and CRISPR/Cas9 screens identified EMC components important for DENV and ZIKV replication[227]. In a separate study SEL1L, UBE2J1, EMC3, EMC2, DERL2, UBE2G2, and HRD1 were reported to trigger WNV-induced cell death without affecting viral replication [233]. The EMC is a membrane protein complex that plays a role in inserting transmembrane domains into the ER membrane [238,239]. Current consensus is that flaviviruses exploit EMC components for different purposes during their viral lifecycle, with the requirements during WNV infection differing from DENV and ZIKV infection. The exact contribution of the EMC complex in the flaviviral lifecycle therefore warrants further investigation, especially during comparative analysis using different flaviviruses.

The signal peptidase complex (SPCS) components were also shown to be important during infection of human (293 T and HeLa) and insect (Drosophila and mosquito) cells, with *SPCS1* important for WNV, DENV, ZIKV, JEV, YFV, and HCV infection. SPCS was implicated in processing of flaviviral structural proteins prM and E. However, whether this is direct or indirect processing warrants further investigation. Similarly, the oligosaccharyltransferase (OST) complex was identified as an important factor that influences DENV replication [227]. A genome-wide CRISPR screen in Huh7.5.1 cells revealed the importance of the OST complex during DENV infection and viral RNA replication, independent of its canonical role in N-linked glycosylation [229]. This is congruent with an independent CRISPR screen to identify host proteins necessary for DENV-mediated cell death in Huh7.5.1 cells [230]. The OST components displayed less pronounced inhibitory effects on YFV, WNV, and ZIKV infection in Huh7.5.1 cells [229,230]; however, they were shown to be required for efficient replication in HeLa cells [240], suggesting a cell-type specific effect.

## Conclusion and future perspectives

Experimental *in vitro* and *in vivo* models have fostered a significant understanding of the balance between flavivirus lifecycle and immunopathology. These studies have established the interplay that exists between the viral lifecycle, host innate and adaptive immunity, that determine severity of disease outcomes. Several important questions regarding flavivirus infection and host interaction remain unanswered, which include the identity of specific receptors for host cell entry, the mechanism of biogenesis of replication organelles, strategies of immune evasion and how the neurotropic flaviviruses cross the blood brain barrier. The advent of better experimental models combined with sophisticated mass spectrometry and proteomics approaches promise to shed light on some of these questions that have remained elusive so far.

## Data Availability

Data sharing is not applicable to this article as no datasets were generated or analysed in the current study. All figures have been deposited in Figshare with DOI 10.6084/m9.figshare.17004592
